# Exploiting hiPSCs in Leber's Hereditary Optic Neuropathy (LHON): Present Achievements and Future Perspectives

**DOI:** 10.3389/fneur.2021.648916

**Published:** 2021-06-08

**Authors:** Camille Peron, Alessandra Maresca, Andrea Cavaliere, Angelo Iannielli, Vania Broccoli, Valerio Carelli, Ivano Di Meo, Valeria Tiranti

**Affiliations:** ^1^Unit of Medical Genetics and Neurogenetics, Fondazione IRCCS Istituto Neurologico Carlo Besta, Milan, Italy; ^2^IRCCS Istituto delle Scienze Neurologiche di Bologna, Programma di Neurogenetica, Bologna, Italy; ^3^San Raffaele Scientific Institute, Milan, Italy; ^4^National Research Council (CNR), Institute of Neuroscience, Milan, Italy; ^5^Department of Biomedical and Neuromotor Sciences-DIBINEM, University of Bologna, Bologna, Italy

**Keywords:** Leber's hereditary optic neuropathy, human induced pluripotent stem cells, mitochondrial disorders, organoids, retinal ganglion cells (RGC)

## Abstract

More than 30 years after discovering Leber's hereditary optic neuropathy (LHON) as the first maternally inherited disease associated with homoplasmic mtDNA mutations, we still struggle to achieve effective therapies. LHON is characterized by selective degeneration of retinal ganglion cells (RGCs) and is the most frequent mitochondrial disease, which leads young people to blindness, in particular males. Despite that causative mutations are present in all tissues, only a specific cell type is affected. Our deep understanding of the pathogenic mechanisms in LHON is hampered by the lack of appropriate models since investigations have been traditionally performed in non-neuronal cells. Effective *in-vitro* models of LHON are now emerging, casting promise to speed our understanding of pathophysiology and test therapeutic strategies to accelerate translation into clinic. We here review the potentials of these new models and their impact on the future of LHON patients.

## Introduction

Leber's hereditary optic neuropathy (LHON) is caused by maternally inherited missense point mutations of mitochondrial DNA (mtDNA) (1) and is estimated as the most-frequent mitochondrial disease (2). This blinding disorder is characterized by selective degeneration of retinal ganglion cells (RGCs), the retinal neurons projecting their axons, which form the optic nerve to the brain. Thus, the extended loss of RGCs and their axons leads to optic nerve atrophy, with a severe defect of central vision, in most cases leaving the patient legally blind (3, 4). Almost all LHON maternal lineages present with homoplasmic mutation (100% mtDNA copies are mutant in all tissues), having one of three frequent mtDNA mutations found in over 90% of patients worldwide (m.11778G>A/*MT-ND4*, m.3460G>A/*MT-ND1*, m.14484T>C/*MT-ND6*), but only some individuals develop the disease. Also, despite that the homoplasmic mtDNA mutation is present in all tissues, only a cellular type, that is, RGCs, undergoes degeneration. The pathogenic mechanism leading to cell death is thus extremely tissue and cell specific (3, 4). The phenotype of these mutations characterized by defective ATP synthesis when driven by complex I substrates (5), increased oxidative stress (6, 7), and increased propensity to undergo apoptosis (8, 9) has been thoroughly investigated in cybrids, lymphocytes, and fibroblasts but not in RGCs, the disease's target, which are not easily accessible and cannot be maintained *in vitro* (10).

Moreover, given the difficulties in manipulating mtDNA, very few animal models with mtDNA pathogenic mutations are available (11), preventing the possibility to study the affected tissues and organs and test therapeutic options.

To overcome these issues, we and other investigators exploited innovative approaches, based on the use of human-induced pluripotent stem cells (hiPSCs) as a faithful source of human neuronal cells and RGCs.

The use of hiPSCs to obtain terminally differentiated cells of a variety of tissues is a revolutionary approach to understanding disease mechanisms, performing drug screening, and testing gene or cell therapy (12–15).

Several studies have demonstrated the possibility to generate neurons and RGCs from plated hiPSC-derived embryoid bodies (16–19). In addition, different groups developed 3D culture systems recapitulating key steps of retinal development and allowing the generation of self-organizing retinal organoids containing RGCs (15, 20–25). These models provide a bridge between traditional 2D cell culture and mouse models, representing a paraphysiologic system with pros and cons (26), but of paramount importance for modeling mtDNA-related disorders.

Modeling LHON mutations in differentiated neurons and organoids will provide not only insights into the tissue-specific disease pathogenic mechanisms, but it will offer the unique opportunity to test *in-vitro* pharmacological approaches in a model system much more relevant than traditional non-neuronal cell cultures, such as fibroblasts, lymphoblasts, or cybrids. Moreover, patient-specific hiPSCs allow studying the effect of the mtDNA mutation in the context of patient-specific nuclear background, which, in LHON particularly, plays a pivotal role in the modulation of disease's presentation (27).

We here discuss our experience with the generation of hiPSCs from LHON-affected patients integrated with the data present in the literature. We particularly emphasize the translational potential for patients in exploiting LHON neuronal cells and RGCs to advance our knowledge of pathogenic mechanisms and test therapies.

## Reprogramming Fibroblasts or Peripheral Blood Mononuclear Cells PBMCS From LHON Patients

Since the epochal discovery of induced pluripotent stem cells by the Yamanaka group in 2006 (28), many researchers generated hiPSC by reprogramming differentiated cells obtained from mitochondrial disease patients [MELAS syndrome (29), MERRF syndrome (30), Pearson syndrome (31); reviewed by Liang (32)]. Unexpectedly, even if LHON is the most-frequent mitochondrial disease, to date only a few groups, including ours (33), had generated LHON hiPSCs by reprogramming fibroblasts or peripheral blood mononuclear cells (PBMCs) derived from patients (34–38).

One group from Taiwan reprogrammed PBMCs from two LHON m.11778G>A patients and one LHON m.11778G>A unaffected carrier using the Sendai virus (37). The authors reported a slightly increased complex I (CI) activity, failing statistical significance, in the LHON hiPSCs, both affected and carrier, as compared to control. The authors reported a slightly increased of complex I (CI) activity in both affected and carrier LHON hiPSCs as compared to control, that failed to reach statistical significance.

Another group reprogrammed fibroblasts from two LHON m.11778G>A patients as well as one LHON proband carrying two mutations m.4160T>C and m.14484T>C, using episomal vectors expressing six reprogramming factors OCT4, SOX2, KLF4, L-MYC, LIN28, and shRNA for p53 (34). They investigated hypothetical difficulties in reprogramming cell lines with OXPHOS defects since Yokota et al. reported reduced reprogramming efficiency in mitochondrial encephalomyopathy with lactic acidosis and stroke-like episodes syndrome (MELAS) fibroblasts carrying more than 90% of the m.3243A>G mtDNA mutation (39). Hung and collaborators reprogrammed fibroblasts carrying the homoplasmic LHON mutations and found no significant differences in the number of hiPSC colonies between controls and LHON patients (21 colonies on average for the controls, and 13 colonies on average for the LHON patients). Differently, our own experience with LHON was more similar to what observed by Yokota, since we noticed that LHON fibroblasts or PMBCs are refractory to be reprogrammed to hiPSC.

**Figure 1:**
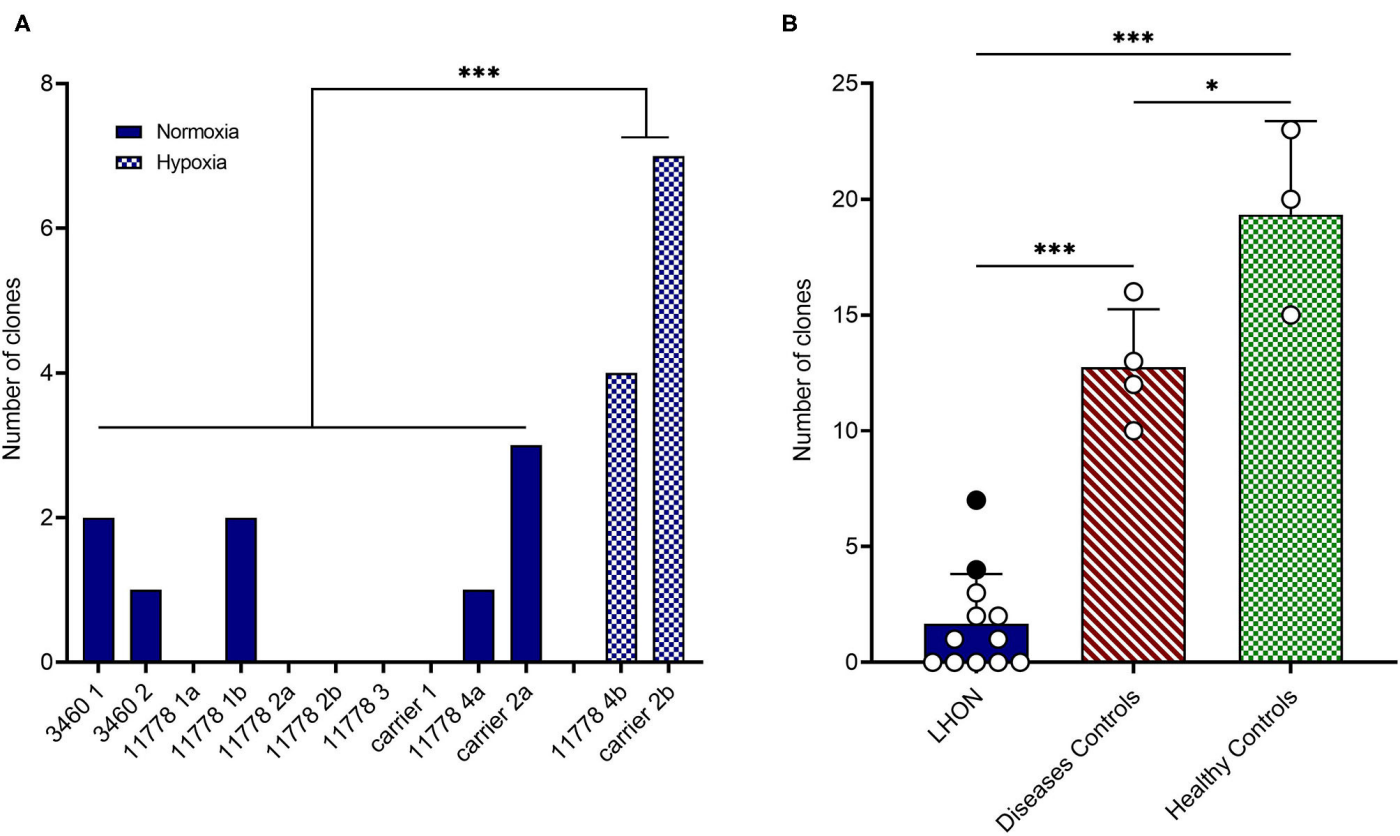
LHON cell lines reprogramming efficiency. **(A)** Number of hiPSC clones obtained by reprogramming affected or non-affected (carrier) LHON-derived fibroblasts or PBMC under normoxia (solid blue bars) or hypoxia (cross-hatched blue bars) conditions. **(B)** Comparison between grouped numbers of hiPSC clones obtained from LHON (solid blue bar), disease controls including non-LHON mitochondrial diseases (hatched red bar), and healthy controls (cross-hatched green bars) cell lines. The dots represent the number of clones obtained under normoxia (white dots) or hypoxia (black dots) conditions. ^***^p < 0.001, ^*^p < 0.05.

Specifically, we attempted to reprogram different LHON cell lines: two m.3460G>A patients, four m.11778G>A patients, and two unaffected m.11778G>A carriers (Table 1). As shown in Table 1 and Figure 1A, the number of clones obtained was in general very low, even if numerous attempts were performed also in different laboratories. Conversely, using fibroblasts derived from healthy controls or disease's patients affected by mitochondrial disorders, including dominant optic atrophy (*OPA1* mutation), Pearson (40), and MPAN (41), we obtained on average from 10 to 20 clones of hiPSC (Table 1 and Figure 1B) per reprogramming experiment. To overcome this issue, we tested the reprogramming efficiency of LHON cells under hypoxia laboratory conditions (5% pO_2_, more similar to physiological oxygen tension *in vivo*), a condition previously used to enhance the generation of hiPSC (42), and recently demonstrated to be specifically beneficial in several OXPHOS defects, by improving disease phenotype in mice and cells (43). In fact, under traditional culturing conditions cellular models of mitochondrial respiratory-chain disease and Friedreich's ataxia showed proliferative defects, which could be reversed by lowering oxygen tension (44). In addition, hypoxia was able to prevent and even reverse the neurological phenotype in a Leigh syndrome mouse model characterized by CI deficiency due to *Ndufs4* gene ablation (45). Based on this evidence and since LHON mutations were associated with reduced CI-driven ATP synthesis and increased ROS production (46), we hypothesized that hypoxic cell culture conditions during reprogramming could increase the number of hiPSC clones generated. Thus, we recently reprogrammed PBMCs derived from one LHON m.11778G>A patient and one carrier, in parallel under normoxic (11778 4a and Carrier 2a) and hypoxic (11778 4b and Carrier 2b) conditions (5% oxygen), following published procedures (42). We found that this hypoxic condition significantly increased the number of hiPSC clones generated (Table 1 and Figure 1A). In fact, while under normoxic conditions, we obtained around nine LHON hiPSCs clones in 10 different reprogramming experiments (0.9 clones/reprogramming cycle), and this number increased, under hypoxic conditions, to 11 clones in two reprogramming experiments (5.5 clones/reprogramming cycle). Although these last results derived from only two experiments and need to be further consolidated, they indicated a statistically significant improvement of the reprogramming efficiency (Figure 1A), which remains largely below that observed for the disease control group (12.7 clones/reprogramming cycle) and for the healthy control group (19.3 clones/reprogramming cycle) (Figure 1B). This amelioration of the reprogramming efficiency is relevant not only to obtain enough biological material for further investigations but could also unravel an insight into pathogenic mechanisms, relevant for the disease, and for the development of targeted effective therapy. Remarkably, the subacute phase of LHON is hallmarked by well-known vascular changes, and ongoing discussions revolve around the issue of pseudo-hypoxic signaling that RGCs may produce as their metabolic unbalance reaches the threshold for triggering the disease, possibly underlying the microangiopathy in LHON (3, 4, 47, 48).

**Table 1:** Characteristics of cell lines subjected to reprogramming.

**Individuals**	**Cell line name**	**Gene mutated**	**Nucleotide change**	**Cell type reprogrammed**	**Reprogramming conditions**	**Clones obtained**
LHON patients	3460 1	MT-ND1	m.3460G>A	FB	Normoxia	2
	3460 2	MT-ND1	m.3460G>A	FB	Normoxia	1
	11778 1a	MT-ND4	m.11778G>A	FB	Normoxia	0
	11778 1b	MT-ND4	m.11778G>A	FB	Normoxia	2
	11778 2a	MT-ND4	m.11778G>A	FB	Normoxia	0
	11778 2b	MT-ND4	m.11778G>A	FB	Normoxia	0
	11778 3	MT-ND4	m.11778G>A	FB	Normoxia	0
	11778 4a	MT-ND4	m.11778G>A	PBMC	Normoxia	1
	11778 4b	MT-ND4	m.11778G>A	PBMC	Hypoxia	4
	carrier 1	MT-ND4	carrier m.11778G>A	FB	Normoxia	0
	Carrier 2a	MT-ND4	carrier 90% m.11778G>A	PBMC	Normoxia	3
	Carrier 2b	MT-ND4	carrier 90% m.11778G>A	PBMC	Hypoxia	7
Disease controls	DOA	OPA1	c.1334 G>A	FB	Normoxia	13
	MPAN	C19orf12	c.172G>A	FB	Normoxia	12
	Pearson 1	mtDNA macrodeletion	m.9449_14550 del	FB	Normoxia	10
	Pearson 2	mtDNA macrodeletion	m.8469_13460 del	FB	Normoxia	16
Healthy controls	Control 1	None	none	FB	Normoxia	23
	Control 2	None	none	FB	Normoxia	20
	Control 3	None	none	PBMC	Normoxia	15

## Generation of RGCs From LHON Patients

In the last years, a few protocols have been developed with the purpose to differentiate RGCs directly from patients-derived hiPSCs. However, very few of these RGCs models have been produced for LHON. The first model was reported in 2017 by the Wong group, who generated RGCs from one healthy control and one patient carrying in combination the two homoplasmic mtDNA mutations m.4160T>C and m.14484T>C. Interestingly, they used cybrid technology to also generate patients' fibroblasts homoplasmic for the wild-type mtDNA, thus creating an isogenic control hiPSCs and derived RGCs (35). They found an increased level of apoptosis in LHON RGCs not observed in the healthy and isogenic corrected RGCs, demonstrating that this phenotype was a direct consequence of the LHON mutations. Another group generated hiPSCs-derived RGCs from a m.11778G>A LHON-affected and unaffected carrier, belonging both to the same family (37). They observed enhanced mitochondrial biogenesis, decreased basal respiration, and increased oxidative stress in both affected and unaffected RGCs. However, defective neurite outgrowth was only found in the affected RGCs, while carrier cells exhibited a prominently higher expression of the gene encoding γ-synuclein. Interestingly, increased CI activity was observed in RGCs derived from the asymptomatic carrier but not from the affected patient. Differences in affected and unaffected RGCs carrying homoplasmic m.11778G>A mutation were also found by Yang et al. (49). Both lines showed increased ROS production, but only the affected cells were characterized by increased apoptosis and altered mitochondrial transport pattern along the axons, with an increase in retrograde and a decrease in stationary mitochondria. Furthermore, affected RGCs displayed a significant increase of KIF5A, a member of the kinesin-1 family KIF5, involved in the transport of mitochondria along the axons. Another study carried out on hiPSC-derived RGCs by Yang et al. (50) highlighted the possible role played by AMPA receptors and excitotoxicity in m.11778G>A LHON patients. They used a modified protocol of differentiation of hiPSCs to RGCs to obtain a highly homogeneous RGCs population. They showed how the *MT-ND4*-mutated LHON-RGC cells exhibited significantly reduced GluR1/R2 (subunits of AMPA receptors) and their associated scaffold proteins and the resulting different pattern of response to glutamate stimulation compared to control.

Lastly, Edo et al. (51) demonstrated that hiPSC-derived RGCs can suppress the immune activity of T-cells via TGF-β, have a poor expression of HLA class I, and no expression of HLA class II (CD80 and CD86 co-stimulatory molecules), opening the possibility of using these cells in transplant without the risk of rejection.

## Generation of Neurons From LHON Patients

Almost two decades ago, the Cortopassi group generated cybrids using the neuronal precursor cell line NT2, containing mitochondria from patients with m.11778G>A and m.3460G>A mutations (52). Differentiation of LHON-NT2 cells resulted in a decreased number of cells, reduction of mtDNA amount, and increased ROS production, compared to the parental line. To our knowledge, no hiPSCs-derived neuronal model different from RGCs has been generated to date. Although it is clear that RGCs represent the best model to unravel LHON pathomechanisms, hiPSCs differentiation in non-RGCs neurons could be informative as well to study the selective degeneration of RGCs in patients. To maintain the transparency of the retina to light, the retinal segment of the RGCs axon is unmyelinated, increasing the energetic demand for action potential firing along this portion and making these cells particularly susceptible to energetic deficit (53). The generation of *in-vitro* myelinated neurons through co-cultures of Schwann cells and hiPSCs-derived neurons (54) might be informative to establish the involvement of myelin in the pathogenesis of the disease.

## State of the Art on Organoids Implementation

The use of 3D organoids generated *in vitro* from patient-derived cells may represent an important interface between *in-vitro* and *in-vivo* modeling of LHON, being more accessible and easier to obtain than mouse models and overcoming the anatomical interspecies differences between humans and rodents.

The first human brain and retinal organoids have been generated about 10 years ago from different groups (55, 56). Lancaster and colleagues successfully modeled genetic microcephaly using hiPSCs derived from patients' fibroblasts to generate brain organoids.

Only a year before, the Sasai group had generated a 3D optic structure by self-organization of cultured human embryonic stem cells (ESCs). The optic cup consisted of the retinal pigmented epithelium, and an inner neural retina correctly organized into multilayered tissue containing photoreceptors (rods and cones), interneuron precursors, and RGCs (57). Both these protocols exploited the capacity of embryoid bodies (EBs) (ESCs or hiPSCs-derived) to proceed spontaneously toward ectodermal commitment without extrinsic signaling factors, which instead are necessary for mesodermal and endodermal specifications (26, 58).

Several modifications and adjustments to the pivotal approaches of Lancaster (55, 59) and Sasai group (57, 60), have been done in the following years, essentially identifying distinct extrinsic factors to obtaining specific regions in the organoids (60, 61), or by-passing the EBs formation step (62). Moreover, improvements toward standardization are constantly evolving, such as the use of completely xeno-free culture methods (62) or the introduction of technologies allowing large-scale controlled organoids production, such as bioreactors or microfluidics chips (26). Importantly, also protocols for cryopreservation at intermediate steps of differentiation have been established, allowing the biobanking of the *in-vitro*-generated organoids, an additional advantage compared to animal models (26, 57, 62).

## Therapeutic Approaches

Despite the numerous clinical and pre-clinical investigations carried out to date, effective therapies for LHON are still limited. Effective means that therapy should be able to tangibly modify the disease natural history either by aborting or reverting the catastrophic wave of cell death, or at least limiting the progression so that the visual function is substantially preserved based on anatomical RGCs measurable sparing. Multiple clinical trials have been conducted in recent years, essentially targeting the main pathways involved in the pathogenic mechanism (63). Several antioxidants molecules, some of which with direct effects on mitochondrial respiration, have been tested in patients: idebenone, Coenzyme Q10 (CoQ10), EPI-743, Elamipretide, curcumin (63).

To date, idebenone (Raxone^®^) is the only drug approved by the European Medicines Agency for LHON. It has been documented that idebenone can increase the rate of visual recovery in LHON patients after reaching a nadir of visual loss (64–66); however, its efficacy remains incomplete and variable amongst treated subjects.

The only treatment explored in LHON hiPSCs-derived RGCs was the antioxidant N-acetyl-L-cysteine, which was shown to reduce the ROS production and apoptosis, also rescuing the defective mitochondrial transport observed in the LHON cells (49).

Additional compounds targeting other pathways involved in the LHON pathogenesis (mitobiogenesis, mitophagy, mitoinflammation) have been evaluated only in patient-derived primary cells or in cybrids, such as phytoestrogens (67), rapamycin (68), papaverine, and zolpidem (69). Moreover, other potential strategies are emerging, for example, the inhibition of the miRNA181a/b, acting on both mitobiogenesis and mitophagy (70). All these pharmacological approaches should be reevaluated also in RGC to understand if they are efficacious and rapidly translatable into a therapy.

Besides pharmacological clinical trials, encouraging results are nowadays being reported by clinical trials with gene therapy for patients carrying the m.11778G>A/*MT-ND4* mutation [reviewed in Amore et al. (63)], using the Adeno-Associated Virus (AAV)-mediated allotopic expression of a wild-type recoded version of the mtDNA-encoded ND4 subunit of complex I (71, 72). To better refine the efficiency of allotropic expression strategy in the context of RGCs, detailing the mitochondrial import of wild-type ND4 protein, its competition with the endogenously expressed mutant ND4, and finally the dynamics of complex I assembly of either one or the other ND4 subunits, may greatly benefit of 3D organoid modeling of LHON. This might resolve some of the criticisms previously raised by the preclinical studies (73–75). The same approach could be developed for the other LHON-related mutations, and different approaches based on gene therapy might be proposed in the future, for example, modulating the expression of modifying genes or miRNAs (70) or applying possible gene-editing strategies, as recently proposed for mtDNA (76). Similarly, the feasibility of mitochondrial import of nucleic acids, claimed by some studies (77, 78), may benefit the use of eye/brain organoids carrying LHON mutations, reproducing those experiments and possibly paving the road for further gene therapy strategies.

## Discussion

*In-vitro* modeling of LHON through 2D cell cultures, including patient-derived hiPSCs and neurons, allowed important steps forward in the understanding of the pathogenic mechanism of this complex and fascinating disease. We here presented evidence that LHON hiPSCs are difficult to obtain as compared to other apparently more severe mitochondrial disorders, but this reduced efficiency could be improved by performing the reprogramming experiment under hypoxic conditions. This observation would deserve further investigation since obtaining a large number of hiPSCs clones is instrumental to further develop differentiated 2D cell cultures. Although 2D cell cultures show several advantages such as easy manipulation and analysis (good accessibility of nutrients and/or drugs, excellent visualization and tracking of cells at microscopy by live-cell imaging), the complex 3D architecture of *in-vivo* tissues is not reproduced by this method, nor are the interactions between different co-resident populations of specialized cells (79). This is particularly important for LHON, in which RGCs are the only cells affected in the retina. The application of the innovative single-cell omics on hiPSCs-derived 3D organoids can provide useful insight on the cell specificity of LHON disease. A recent study has already paved the way for this approach, performing single-cell transcriptomics on *in-vitro*-generated human retinal organoids and *ex-vivo* adult human retinas, allowing mapping of disease-associated genes to particular cell types (25). This work highlights the importance of investigating mechanisms of disease in RGCs since they could be differently regulated in the traditional cell models so far exploited. Many of the findings so far achieved in LHON should be revalidated in RGC models to assure that the right pathogenic mechanism was effectively targeted by therapies.

Modeling mitochondrial diseases caused by mtDNA mutations in animals is still challenging due to the difficulties in manipulating the mitochondrial genome (80, 81), although a new promising method has been recently described Mok et al. (76). In 2012, the group of Doug Wallace, a pioneer in the field of mitochondrial medicine, successfully generated a mouse model carrying a mutation in the *MT-ND6* gene, which developed a pathology closely resembling LHON at 2 years of age, although the mouse did not show reduced visual responses (82). This model was instrumental to reproduce some of the hallmark features observed in human post-mortem LHON retina (83, 84); however, mice, because they lack the macular region, ultimately fail to reproduce the natural history that clinically characterizes humans with the characteristic catastrophic evolution of RGC neurodegeneration (3, 4).

Thus, it will be fundamental to investigate pathogenic mechanism of LHON disease in hiPSCs-derived cell/tissue-specific models and retinal organoids might be instrumental to assess efficacy/toxicity in the pre-clinical phases. The issue of maintaining organoids in a spinning bioreactor under hypoxic conditions, with the intent of reproducing the brain endogenous developmental program, could be crucial, especially for LHON in light of our observation, but also in general for other diseases. To date, only a few brain organoids models of mitochondrial diseases have been reported, specifically for MELAS syndrome, mitochondrial neurogastrointestinal encephalomyopathy, Friedrich ataxia, and Leigh syndrome (85–88). We think that modeling LHON with retinal organoids would provide substantial progress in the understanding of the pathogenic mechanisms and in identifying the correct targets for therapy development. To this end, testing pharmacological and gene therapy approaches with human transgenes packaged in the appropriate AAV vector constructs, currently performed in animal models with obvious problematic issues (89, 90), may benefit human-patient-derived eye/brain organoids, certainly allowing to speed translation from pre-clinical science to approval for human clinical trials of regulatory agencies such as the Food and Drug Administration (FDA) and European Medicines Agency (EMA).

## Data Availability Statement

The raw data supporting the conclusions of this article will be made available by the authors, without undue reservation.

## Ethics Statement

The studies involving human participants were reviewed and approved by Fondazione IRCCS Istituto Neurologico Carlo Besta. The patients/participants provided their written informed consent to participate in this study.

## Author Contributions

CP, AC, AI, and ID perform experiments, analyzed data, generated the table and figure, and analyzed the literature. AM, VB, and VC analyzed the literature. VT concept the manuscript architecture, supervised the analysis of the data, and of the literature. VT performed the final revision of the manuscript. All the authors draft the manuscript.

## Conflict of Interest

The authors declare that the research was conducted in the absence of any commercial or financial relationships that could be construed as a potential conflict of interest. The handling editor is currently organizing a Research Topic with one of the author VC.
